# Let the Farmers Embrace “Carbon Neutrality”: Taking the Centralized Biogas as an Example

**DOI:** 10.3390/ijerph19159677

**Published:** 2022-08-05

**Authors:** Qiang Wang, Liying Yu, Yueling Yang, Haoran Zhao, Yanqing Song, Wenhao Song, Jinmeng Liu

**Affiliations:** 1 School of Business, Shandong Management University, Jinan 250357, China; 2 School of Humanities, Shandong Management University, Jinan 250357, China

**Keywords:** carbon neutrality, sample selection model, willingness to embrace (WTE), willingness to motivate (WTM)

## Abstract

The promotion of rural centrally produced biogas (CPB) is an effective carbon neutrality development solution in rural areas. How to better encourage farmers to adopt such products is an important part of the sustainable development of a project. For this reason, focus is needed on the “willingness to embrace (WTE)” and “Willingness to motivate (WTM)” of rural residents for CPB projects and their influencing factors. We chose to conduct questionnaire surveys in rural areas of the Hebei and Shandong provinces of China, using the contingent valuation method (CVM). The results show that 85% of the respondents support CPB. Compared with urban gas, the subsidy demand of rural residents for CPB is 56.78%. The influencing factors of the residents’ WTE are affected by the number of children in the family, whether the village cadres are installed in the family, solar water heaters installed in the family, knowledge and attitudes towards environmental protection, and the embracing of daily energy habits. The influencing factors on the residents’ WTM are age, education level, ownership of arable land, knowledge of environmental protection, etc. Therefore, we propose policy recommendations. First, we must fully understand the willingness and demands of farmers, adopt a reasonable compensation response mechanism, and scientifically calculate financial inputs. The second step is to guide farmers through multi-channel publicity. Third, we aim to improve project operation efficiency, reduce operating costs, and minimize the government’s financial burden on the basis of ensuring that farmers’ demands are considered in a coordinated manner.

## 1. Introduction

On 22 September 2020, the Chinese government made it clear at the 75th United Nations General Assembly that it will strive to reach peak carbon emissions before 2030, and strive to achieve carbon neutrality by 2060. Carbon emissions will be an important binding indicator for China’s social and economic development in the future [1]. Carbon reduction in vast rural areas is an important part of China’s goal of achieving carbon neutrality. Biomass energy is a renewable energy with natural energy storage features, carbon neutrality and stable output guarantee. Under the background of China’s “carbon peak, carbon neutrality” goals and rural revitalization strategy, the rural energy sector needs to undergo fundamental changes. It is urgent to develop advanced technologies, establish a low-carbon and clean energy system, and solve rural energy and environmental problems [2]. Therefore, it is of far-reaching significance to study the establishment of a low-carbon and clean energy system in rural areas and to develop related technologies.

Rural biogas is an effective means for farmers to embrace “carbon neutrality” by improving the rural energy mix. One household can reduce about 2.0 t CO_2_ equivalent pollutants annually by consuming 275 m^3^ of biogas [3]. Large and medium-sized centrally produced biogas (CPB) projects in rural areas use agricultural waste as the main anaerobic fermentation raw materials to produce biogas and provide a unified supply for the community to improve the quality of life of rural residents [3]. This type of project has a good environmental protection effect, and can reduce air pollution by directly reducing the open burning of straw and the burning of scattered coal in rural areas [4]. It can effectively control air pollution and reduce CO_2_ emissions [5]. China’s current mature straw biogas projects are mainly medium-scale [6].

China‘s rural biogas is changing from distributed to centralized. The traditional rural household biogas is mainly invested in by farmers, and the government subsidizes part of the construction funds. Farmers own the property rights. The investment subject of CPB is diversified. There are also various modes of investment and operation of the project [7]. The Chinese government’s support policy for rural biogas projects has changed. It no longer subsidizes household biogas with relatively low operating efficiency, but instead supports large and medium-sized biogas projects that are mainly centralized [8]. After practical tests, large and medium-sized projects have obvious advantages in terms of economy, ecology, environment, and social benefits [9]. The Chinese government plans to use about 25 billion m^3^ biomass gas annually by 2035 [10].

As consumers, how to better encourage farmers to adopt CBP is an important part of the sustainable development of a project. Due to the diverse investment and operating models of CPB, consumers may not be limited to farmers. In this research, the main emphasis is on the CPB projects built in rural communities with the dual characteristics of public welfare and profit, with farmers as the main consumers. Biofertilizers formed from residues such as biogas slurry and biogas residues are also marketed. The rural CBP can provide farmers with high-quality living energy, optimize the rural energy consumption structure, reduce greenhouse gas emissions, and promote low-carbon economic development [11]. Biogas slurry and biogas residue are also very high quality organic fertilizers. These organic fertilizers improve the quality of agricultural products, and promote the development of organic ecological agriculture and the circular economy [12]. It can be called a multi-win, and it is an important starting point for agricultural transformation and upgrading. It is necessary to explore the rural biogas subsidy policy and compensation mechanism. In particular, research should explore the subsidy transition from the current biogas construction investment to the key links of the whole industry chain, especially the front-end raw material subsidy and the back-end product and user use subsidy policy. At present, developed countries implement tax exemptions or tax reductions for the use of biogas, and provide incentives such as subsidies for the development of biogas. However, China has no economic policy support for raw material production, collection and transportation, and biogas end-users [13].

The premise of enabling farmers to better embrace “carbon neutrality” is to fully understand farmers’ economic demands in order to cope with changes to farmers’ lives, according to the theory of ecological compensation, with the aim of protecting sustainable utilization of ecosystem services, mainly for economic means, adjust the interests of stakeholders, and finally realize various rules that can promote compensation activities and mobilize the enthusiasm for ecological protection [14]. Because farmers are consumers, are they willing to embrace this? What is the level their willingness to be motivated? Which influencing factors can improve farmers’ willingness to embrace (WTE) and their willingness to motivate (WTM)? These questions will affect the sustainability of the project [15]. WTE questions whether, if a CPB station is established and rural residents as users, do they intend to accept and adopt the generated biogas? WTM suggests that if they are willing to accept and adopt centralized biogas, what are their motivational demands? Although the quality of life is improved, it also increases the cost of living, so what percentage of subsidy do they want the government to give? Therefore, it is necessary to investigate the willingness of rural residents to achieve the sustainable development of the project [16].

Contingent Valuation Method (CVM) refers to a method that mainly uses questionnaire surveys to directly investigate the economic behavior of respondents in a hypothetical market, and measures the value of goods or services by obtaining consumers’ willingness to pay [17,18]. CVM is the most widely used and most influential method in non-market valuation techniques. It mainly uses questionnaires to directly examine the economic behavior of respondents in a hypothetical market, so as to obtain information on consumers’ willingness to pay to measure the value of goods or services [19]. It assumes that the decision-making of rural residents is reasonable, the maximization of utility is the behavioral goal of rural residents, and the expected quality of life becomes the basis of their behavioral decision-making [20].

At present, there is insufficient research on the needs and willingness of users for CPB projects. This article will understand the market demand of CPB projects from the perspective of users and provide reference for decision makers. Therefore, we will investigate and analyze farmers’ needs and perceptions of such “carbon neutral” projects, understand their WTE and WTM, and what factors can improve farmers’ WTE and WTM. So, taking WTE/WTM as dependent variables, and multiple causal attributes as independent variables, we will find the causal relationship among them, improve WTE/WTM through intervention, and finally increase the depth of farmers’ embrace of “carbon neutrality”. This is the novelty and originality of this article and the main contribution of this work.

## 2. Methods and Data

### 2.1. Investigation Regions

**Figure 1:**
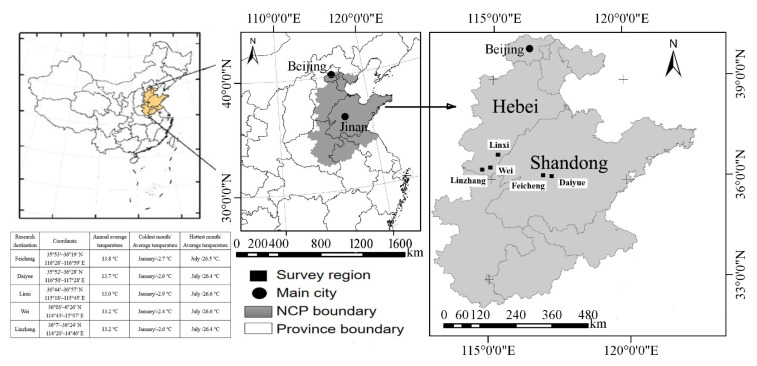
The investigated regions.

The scope of the investigation has been restricted in Hebei and Shandong provinces (Figure 1) for these reasons. In these areas, agricultural production and migrant employees are the biggest sources of financial gain for rural residents in these areas, and therefore the level of economic and social development is roughly similar. The weather conditions and energy habits are similar, the rural population is densely populated, and a large amount of scattered coal is used. It used to be the most polluted area in China [21]. They are also major grain producing areas, and the cultivation of corn and wheat makes straw resources rich [22]. These two provinces are also relatively concentrated areas for CPB projects in China [23].

The data was collected through questionnaire surveys conducted in the suburbs of Feicheng City and Daiyue District in Shandong Province, and the suburbs of Linxi County, Weixian County and Linzhang County in Hebei Province from July to November 2018. The selected five regions all belong to the warm continental monsoon climate zone with four distinct seasons (Figure 1, data from the official website of the government).

### 2.2. Investigation Method

We use a fixed-point random sampling survey method. At the survey site, the researcher will first introduce the CPB project to the interviewee within 5 min. The questionnaire uses the double-boundary dichotomous questionnaire method [24]. Each questionnaire takes 20 min. The research team organized the local rural residents to conduct this investigation by contacting the local agricultural department.

### 2.3. Variable Definitions

The variables set in the survey questionnaire are shown in Table 1.

For the dependent variable, we select WTE/WTM. respectively. During the actual investigation process, the first question was “If a centrally produced biogas project is established in the village to provide the residents with biogas, would you like to embrace the produced biogas?”, and the answer options are “1. Yes; 0. No”. If the respondent answers “yes”, then enter the WTM question; if the answer is “no”, then terminate the survey after asking the reason.

Before inquiring about the WTM, the investigator will first explain that “the current municipal gas price is about RMB ¥ 3 per m^3^. With reference to the current average natural gas embrace cost of Chinese urban residents, it is about RMB ¥ 180 per capita per year (survey data, urban residents in the North China Plain) The average natural gas embrace is about 60 m^3^ per year per capita, and the price is RMB ¥ 3 per m^3^).

If one uses CPB and completely gives up using coal and wood, this is equivalent to contributing to the country’s low-carbon sustainable development. The country will give certain subsidies. Compared with the price of urban gas, what proportion of the subsidy do you think is appropriate? What percentage do you want to subsidize biogas prices? The answer options are: “0. No subsidy; 1. 10% subsidy; 2. 20% subsidy; 3. 30% subsidy; 4. 40% subsidy; 5. 50% subsidy; 6. 60% subsidy; 7. subsidy 70%; 8. 80% subsidy; 9. 90% subsidy; 10. 100% subsidy”.

Independent variables mainly come directly from the inquiries of the respondents. It should be noted that “knowledge, attitude, practice” is a behavioral intervention theory: “understanding of any given subject (knowledge)”, “feelings and predetermined opinions about the subject (attitude)”, “the way they demonstrate their knowledge and attitudes through action (practice)” [25,26]. The survey of environmental knowledge variables asked 10 questions to each respondent, with a total score of 10 points. The environmental attitude variable asked respondents about the importance of environmental protection relative to income and health. The total score is 10 points. For example, if the environmental protection score is 3 points, then the attitude score of the respondents is 3 points. Household energy preference is a proxy variable. Mainly clean energy, such as liquefied petroleum gas, electricity, etc., is defined as 1; traditional energy, such as coal, firewood, etc., is defined as 3; when clean energy and traditional energy are divided equally, it is defined as 2.

### 2.4. Descriptive Analysis

From the survey, we obtained 389 questionnaires. After the integration and statistics of the questionnaires, we obtained 351 valid questionnaires. The efficiency of the sample is also very satisfactory, reaching 90.23%. For descriptive statistics, see Table 2 below.

From the current status of household energy use in the surveyed area, 58.97% of households have solar energy, and only 10.54% of households use small-scale biogas. Sadly, most of these are obsolete.

Chinese farmers use hot water frequently in their daily life: by liquefied gas 7.41%, by electric water heaters 11.95%, by coal 51.00%, by firewood 11.68%, and by biogas only 2.28%.

As far as WTM is concerned, when asked if they were willing to build a biogas station to provide residents with enriched biogas, 85.19% of the respondents answered that they are willing to embrace biogas. However, although, so many residents are willing to embrace it, there are still 16.39% of residents worried about the cost. This reflects that, if the cost is affordable, rural residents are very enthusiastic about participating in CPB. Some residents are still worried about the cost, which cannot be ignored. For users who are unwilling to embrace it, 61.54% said they are worried about the cost, so cost is important. The other 38.46% of residents said that they have become accustomed to their current lifestyle and do not want to change.

Regarding the source of cognitive information, nearly a half of respondents, 48.22%., chose TV/newspapers/books; nearly 1/5 respondents prefer village committees. The other figures are: mobile and internet 13.96%; relatives and friends 4.57%; agricultural resource distribution quotient 4.06%; agricultural technology departments 4.31%.

### 2.5. Selection of Measurement Model

The behavior of farmers’ WTE is a binary variable, so the Logit model should be used. The probability of choosing willing to consume is P, and the probability of choosing unwilling to consume is 1−P, so the ratio of the probability of willing to consume and unwilling to consume is $\frac{P}{1-P}$, and the model can be constructed by Equation (1):

(1) $$Y_{WTE}=\ln\left(\frac{P}{1-P}\right)=\beta_{O}+\beta_{1}X_{1}+\cdots +\beta_{15}X_{15}+\varepsilon$$

In Formula (1), $\beta_{O}$ represents the regression intercept, ε is the random disturbance term, $X_{1}$, $X_{2}$…$X_{15}$ represent the explanatory variables, and $\beta_{1}$, $\beta_{2}$…$\beta_{15}$ are the regression coefficients of the corresponding explanatory variables.

The dependent variable $Y_{2}$ is the government’s subsidy ratio for the farmers who are willing to consume, and it is a restricted dependent variable, so the Tobit model is adopted, as in Formula (2):

(2) $$Y_{i}^{*}=\alpha +\sum \beta_{i}X_{i}+\mu_{i}$$

$$Y_{2}=\left\{\begin{array}{c} Y_{i}^{*},\;if\;Y_{i}^{*}>0\\ 0,\;if\;\;Y_{i}^{*}\;\leq 0 \end{array}\right.$$

$Y_{i}^{*}$ is the latent variable; $Y_{2}$ is the observed dependent variable, which represents the desired government subsidy ratio to the price; $X_{i}$ is the independent variable, and $\beta_{i}$ is the correlation coefficient; α is the constant term; $\mu_{i}$ is the random error term.

This research needs to understand the government’s subsidy ratio for products that farmers hope to obtain. First, it needs to understand the farmers’ willingness to consume the products, and further analyze related influencing factors for samples with intentions to embrace. Since it is difficult to observe the willingness and influencing factors of the farmers who chose unwilling to consume, only the willingness and influencing factors of the subsidies are obtained from the farmers who are willing to consume, and there will be sample bias, i.e., in Equations (1) and (2) endogenous problems may appear caused by sample selection problems. Therefore, the Heckman sample selection model is further used for estimation to make the model result more robust [27].

The first stage of the Heckman model is a Probit model containing a full sample, which is used to estimate the probability of the number of people willing to consume in order to solve the problem of missing variables. Specifically, since whether they are willing to consume is a binary variable, a Probit model is established to estimate whether farmers are willing to consume, and the inverse Mills ratio λ is estimated for each sample, i.e., it is calculated for each sample to modify the sample selection of the value of the deviation.

Assuming that farmers are willing to consume CPB products, the Probit model is:

(3) $$Pr(y=1)=\emptyset \left(\beta_{0}+\beta_{1}\overset{n}{\underset{i=1}{\sum }}\beta_{i}X_{i}\right)$$

The left side of Equation (3) is the dependent variable, which represents the probability of a certain event. In this paper, it represents the probability that the farmer is willing to consume (y=1, means that the farmer is willing to consume; y=0, means that the farmer is not willing to consume). The right side of the formula $\emptyset \left(•\right)$ is the cumulative normal distribution function, $\beta_{0}$ is a constant term, $X_{i}$ is an explanatory variable that affects the embrace of products by farmers, and $\beta_{i}$ is the corresponding parameter to be estimated, reflecting the effect of explanatory variables on the extent of farmers’ willingness to embrace. The inverse Mills ratio λ is obtained from the estimation result of the Probit model:

(4) $$\lambda =\frac{\varphi \left(\beta_{0}+\beta_{1}\sum_{i=1}^{n}\beta_{i}X_{i}\right)}{\phi \left(\left(\beta_{0}+\beta_{1}\sum_{i=1}^{n}\beta_{i}X_{i}\right)\right)}$$

In Formula (4), the numerator is the density function of the standard normal distribution, and the denominator is the cumulative distribution function.

In the second stage of the model, the OLS regression method is used to estimate the subsidy ratio that farmers want from the government. In this stage, the inverse Mills ratio λ needs to be included as a correction term along with other variables into the original regression model and the regression parameters are estimated. If the inverse Mills ratio λ parameter is not significant in the second stage, it means that there is no selection bias in the initial regression equation, and it can be estimated directly by the OLS method. Otherwise, it means there is a sample selection bias, and Heckman sample selection model should be used to revise the model.

Substituting λ into the equation of farmers’ willingness to subsidize, the second-stage equation is obtained as follows:

(5) $$Y_{WTM}=\alpha_{O}+\alpha_{1}+\overset{n}{\underset{i=1}{\sum }}\alpha_{i}X_{i}+\omega \lambda +\varepsilon$$

In Formula (5), Y represents the rate of subsidy that farmers want the government to provide for the product; $X_{i}$ is the influencing factor variable that affects the willingness of farmers to subsidize the rate, $\alpha_{O}$ is the regression constant term, $\alpha_{1}$, $\alpha_{i}$, ω are the parameters to be estimated of the corresponding explanatory variables, and ε is a random disturbance term.

## 3. Results

### 3.1. Measurement Results

This paper uses the Stata16.0 software to run the Heckman sample selection model. Finally, we estimate the farmers’ WTE and WTM, and the corresponding influencing factors. The measurement results display that χ = 40.65, *p* = 0.0004. The inverse Mills ratio *λ* is significant, and bias of sample selection exists. Therefore, it is appropriate to use the Heckman sample selection model (Table 3).

### 3.2. WTM Calculation Results

Compared with traditional polluted coal and fuelwood, the cost of CPB has increased. The government guides the vast number of rural residents to use CPB and reduces the burden on residents by subsidizing users, thereby promoting the “removal of coal” and “firewood” in rural areas. According to Formula (5) and Table 3, after eliminating sample selection errors, the expected value of farmers’ WTM for CPB is 56.78% of the expected government subsidy for biogas.

### 3.3. Results Analysis

The influence of individual characteristics: The gender of the respondents is not significant for WTE/WTM, indicating that gender differences are not relevant to CPB adoption and motivational demands. Respondent’s age and education level were not significant for WTE, but significant for WTM. this shows that the age and education level of the respondents have no correlation with the adoption of CPB, but there is a clear positive correlation with the government’s incentive demands. Respondents’ demands for motivation increase as they age. In areas with severe aging, the burden of promoting such projects is heavier. However, as educational attainment increased, so did respondents’ demand for incentives. Through the key interviews in the survey, we also learned that most of the farmers with a high education level have completed the electrification upgrade. The willingness to further improve lifestyle is not high, so the incentive appeal is higher.

The influence of family variables: Variables such as the number of family members, the number of elderly people over the age of 65 in the family, whether there are relatives in the environmental protection department in the family, and family expenditure are not related to WTE/WTM. The area of arable land owned by households is not related to WTE. However, the area of arable land is positively correlated with the incentive demands, but the coefficient is too small, so the influence of arable land area on the incentive demands is small. The number of children under the age of 10 in the household was positively associated with WTE, but not with WTM. This shows that, the more children in the family, the more they want to adopt CPB. However, the number of children does not directly affect the incentive appeal. Families with village cadres are positively related to WTE, indicating that, under China’s national conditions, village cadres support national policies. However, the motivational demands of families on village cadres are irrelevant, and their attitudes are rather ambiguous.

The impact of life energy variables: Households that already have solar water heaters have stronger WTE. In the investigation, we also learned that the installation of solar water heaters is basically an active installation, and there is a demand for energy cost performance. However, the incentive appeal of households installing solar water heaters is not significant. Neither WTE nor WTM was significant for households with domestic biogas installed. During the survey, we also learned that most of the domestic biogas households in China were built under government subsidy and, with the increase of maintenance costs, most of them were abandoned.

The effect of behavioral intervention variables. The environmental protection cognition variables are very significant for WTE/WTM, indicating that the improvement of environmental protection cognition can significantly increase farmers’ willingness to adopt and reduce the incentive cost. Environmental attitudes are only significant for WTE, but not for motivational demands. This shows that the more farmers attach importance to environmental protection, the more they want to adopt CPB, and the incentive appeal is not obvious. The variable of household energy use practice is negatively correlated with WTE, indicating that, in the current household practice, the higher the proportion of clean energy, the stronger the willingness to adopt. However, the incentive appeal is less obvious.

## 4. Discussion

Enabling farmers to embrace carbon neutrality requires the provision of adequate economic incentives. The corresponding subsidy incentive mechanism based on consumer demand is the most important part of the subsidy in the entire biogas industry chain. Demand response is the most cost-effective means [28]. Li et al. give suggestions for the government to set forth the requirements for subsidy to promote green technology or reduce emissions [29]. Wang et al. found the same phenomenon according to the survey of Chinese farmers’ willingness to buy photovoltaics in their homes [30]. The results show that the average maximum willingness of the rural residents in the survey area to be compensated is far from the cost of the current rural CPB project in the survey area. Therefore, in order to realize sustainable development of the project, it is necessary to explore the whole process support from construction to operation. Especially in operation, the incentive mechanism of end users needs to fully understand the demands of users in order to maximize the effectiveness of the compensation mechanism.

More precise incentives are needed for farmers to embrace carbon neutrality. From decentralized to centralized upgrades, the incentive mechanism has become more precise. Consumers’ demands for compensation are inherently price-sensitive [31], and it is necessary to take the terminal market as a breakthrough, adjust the structure of financial subsidies, increase the types of subsidies for terminal products, and improve product price leverage. The pricing mechanism for waste treatment should be further improved, and centralized biogas projects should be included in the catalogue of government environmental protection subsidies, atmospheric governance and ecological compensation [32]. The environmental protection subsidies for waste disposal of relevant enterprises should be increased, and the cost of raw material procurement and disposal of enterprises reduced [33]. Subsidies will be given for the purchase and storage of raw materials for centralized biogas projects that exceed a certain scale, and a government pricing purchase system will be implemented according to the market, region, and type of raw materials [34].

The flexibility of farmers’ willingness will be an important breakthrough in improving the marketization of projects. Through the research results, it can be seen that measures can be taken from different angles to reduce the willingness of farmers to compensate, so as to reduce the government’s incentive cost and improve the marketization level of the project. Wang et al. found that information intervention can directly enhance residents’ waste sorting willingness by researching the impact of information intervention on residents’ willingness to sort municipal solid waste [35]. The government should expand the scope of subsidies for clean energy products, increase subsidies for farmers to purchase centralized biogas products, and reduce the cost of farmers’ purchases. In the promotion process, media such as TV, radio and other windows should be used to enable rural residents to understand environmental protection, clean energy, etc., to understand the role of the application of centralized biogas in protecting the ecological environment, to enhance their environmental responsibility through publicity and education, and to enhance the ecological environment values of rural residents. At the same time, grass-roots village cadres or farmers with strong environmental protection awareness should be guided to first use, and a good atmosphere should be formed through the demonstration effect, so as to encourage more farmers to embrace “carbon neutrality” through the application of centralized biogas.

The various carbon neutrality development solutions currently being promoted in rural areas in China all need to fully consider the farmers’ willingness. In addition to CPB, low-carbon solutions currently being promoted in rural areas in China include biomass briquette [36], biomass power generation [37], distributed photovoltaic power generation [38], small hydropower [39], small wind power [40] and other projects. Farmers are the customers of these projects, and they may also be investors. Customers’ willingness, potential market demand and investment needs need to be fully understood in order to more accurately measure the return on investment of the project [41], as well as the government’s financial responsibility in project investment [42]. The vast rural areas of China are an important support for China’s carbon neutral strategy. Reducing carbon sources and increasing carbon sinks all require the participation of farmers. The WTM of the CPB project is just an introduction. Low-carbon development in rural areas, increasing the enthusiasm of farmers, and intervening in the factors that affect the enthusiasm of farmers will be an important low-carbon development topic [43].

Non-economic incentives such as raising farmers’ awareness are important breakthroughs for farmers to embrace “carbon neutrality”. A very unexpected finding is that, according to our preliminary judgment, households with household biogas have good cognition, so this should be related to WTE and WTM, but it is not actually related. After in-depth understanding during the investigation, we learned that small-scale household biogas projects in rural areas are driven by government subsidies [44], but due to high operating costs, most of them have been abandoned [45]. This also shows that financial support for the construction process is only part of the project promotion process, and sustainable daily operations are more important [46,47]. Therefore, it is necessary to fully understand the active demand behavior of users based on WTE/WTM, and distinguish between passive demand behaviors. Especially, non-economic factors should be identified so as to guide user needs and achieve the sustainable development of the project.

Not only farmers, but all citizens accepting CPB will be an important “carbon neutral” energy solution in the future. In both upgraded and liquid form, it can be used in the transport and industry sector as a fossil-free energy source. First, biogas is of great importance to the automotive industry. Purified biogas will be an important natural gas alternative. The willingness of the transport and industry sector is an important factor in product promotion. Secondly, stakeholders’ perception needs to realize the importance that biofertilizer has for agriculture. The residual product from biogas production can streamline harvests and contribute to environmentally better fertilizers. It is necessary to increase the awareness of the product among all parties, thereby increasing the added value of the product. Third, further studies on citizens’ “carbon neutrality” adoption behaviors can also apply the method constructed in this paper. A “carbon neutrality” strategy is an action that requires active participation from farmers and citizens.

## 5. Conclusions

This paper takes the promotion of centralized biogas as an example, and examines specific incentives for farmers to embrace carbon neutrality. Through surveys in Hebei and Shandong provinces, we found that 85% of respondents support CPB projects. From the perspective of residents’ demand for biogas price subsidies, compared with urban natural gas, the subsidy demand of rural residents for CPB is 56.78%. Therefore, it is necessary to increase the government’s financial investment in such projects and, through sufficient economic incentives, encourage farmers to better participate in the construction of “carbon neutrality”. At the same time, it is necessary to strengthen guidance by intervening in some non-economic factors, such as improving farmers’ knowledge and attitude towards environmental protection, guiding farmers’ energy use habits, and ultimately bringing changes to farmers’ needs. On the basis of fully understanding the needs of residents, there is a need to establish an economic incentive mechanism, correctly calculate the incentive strength, such as a reasonable subsidy rate, and strive to find a sustainable operation mechanism for various “carbon neutral” projects.

In order to enable farmers to more strongly embrace and support carbon neutral strategies, it is necessary to fully understand the willingness of rural residents. To this end, this article puts forward the following policy recommendations: First, we must fully understand the willingness and demands of farmers, reduce costs and increase efficiency, and scientifically calculate financial investment. Only when farmers as consumers are truly involved can their willingness to participate and consumption be stimulated. If farmers become the supporters and beneficiaries of the project, the project will have the impetus for sustainable development. Of course, as a project with both public welfare and profit, under the premise that it is difficult to maintain full marketization, the accounting of financial investment needs to consider various factors such as project cost, farmers’ willingness and the rate of return of investors. The second is to guide farmers through multi-channel publicity. With the improvement of farmers’ knowledge and attitudes, farmers will be more effectively encouraged to participate in “carbon neutrality”. Through publicity and guidance, farmers’ willingness to participate can be effectively improved and financial investment can be reduced. Aiming at factors that can affect the willingness to adopt and to motivate, through multi-channel publicity and guidance, is the right medicine. Third, there is a need to improve project operation efficiency, reduce operating costs, and minimize the government’s financial burden on the basis of ensuring that farmers’ demands are considered in a coordinated manner. With “carbon neutrality” as a hot spot of economic development, the iteration speed of technologies and models is getting faster and faster. The operational efficiency of the project itself is most important. It will be the ultimate goal of the project to maximize the profitability of the project on the basis of the attributes of public welfare and to maximize the reduction of financial investment.

This study has certain limitations and needs further research. First, the sample size is limited. The surveyed farmers are mainly located in parts of the North China Plain in northern China. More comprehensive results will be obtained as the sample is enlarged. Second, the project needs to be further expanded. This paper takes CPB as a typical case of farmers embracing “carbon neutrality”, and further research is needed on the acceptance of farmers for other projects of carbon reduction and sequestration.

## Figures and Tables

**Table 1:** Definition and interpretation of variables.

Variable		Code	Interpretation
Dependent variables		WTE	If there is a centrally produced biogas station, would you like to embrace the produced biogas?
		WTM	If you are willing to consume it, how much do you want the government to subsidize?
Independent variables	Individual characteristic	Gen	Male or female
		Age	Respondent’s age
		Eduyears	Years of education
	Family characteristics	Popu	Number of family members
		Land	Cultivated land area owned by households
		Child	Number of children under 10
		Elder	Number of elderly over 65
		Leader	Is there a village cadre in the family
		Earning	Household income in 2017
		Expen	Household expenditures in 2017
	Household energy embrace	Ifsolar	Is there a solar water heater?
		Ifbiogas	Is there household biogas?
	Behavioral intervention characteristics	Knowl	Understanding of environmental protection topic
		Attit	How it feels and opinions about reservations
		Pract	The way they show their knowledge and attitude through their actions

**Table 2:** Descriptive statistics.

Code	Setting Value	Mean Value	Standard Deviation
WTE	Yes is 1	0.85	0.36
WTM	Integer percentage fron 0% to 100%	5.25%	0.02
Gen	Male is 1	0.59	0.49
Age	years	50.17	11.45
Eduyears	years	7.43	2.98
Popu	Quantity	5.78	2.16
Land	Unit: Mu (666.67 m^2^/Mu)	12.90	41.74
Child	Quantity	1.26	0.99
Elder	Quantity	0.73	0.79
Leader	Yes is 1	0.17	0.38
Rela	Yes is 1	0.04	0.20
Expen	Quantity	20,478	14,851
Ifsolar	Yes is 1	0.59	0.49
Ifbiogas	Yes is 1	0.11	0.31
Knowl	Score	4.98	3.16
Attit	Score	3.09	2.12
Pract	Focus on clean energy: 1; Half clean energy and Half traditional energy: 2; Focus on traditional energy: 3	1.95	0.74

Note: The WTM value of the sample data is the average percentage, that is, the percentage of subsidies that the government can provide. 0. 0; 1. 10%; 2. 20%; 3. 30%; 4. 40%; 5. 50%; 6. 60%; 7. 70%; 8. 80%; 9. 90%; 10. 100%.

**Table 3:** Measurement results.

Cla.	Var.	WTE		WTM	
		Coef.	S. Error	Coef.	S. Error
Individual characteristics	Gen	0.18	0.24	−0.29	0.20
	Age	−0.02	0.01	0.03 ***	0.01
	Eduyears	0.03	0.04	0.09 **	0.04
Family characteristics	Popu	−0.10	0.06	−0.09	0.06
	Land	0.00	0.01	0.00 *	0.00
	Child	0.39 ***	0.14	0.08	0.14
	Elder	0.01	0.14	0.16	0.12
	Leader	0.67 *	0.40	0.34	0.26
	Rela	−0.34	0.74	−0.29	0.48
	Expen	0.00	0.00	−0.00	0.00
Life energy characteristics	Ifsolar	0.53 **	0.23	0.28	0.21
	Ifbiogas	−0.53	0.43	−0.03	0.31
Behavioral intervention characteristics	Knowl	0.13 ***	0.05	−0.09 **	0.04
	Attit	0.23 ***	0.07	0.07	0.05
	Pract	−0.41 **	0.17	−0.15	0.15
	_cons	1.21	0.84	3.74 ***	0.68
	Mills(lambda)			1.48 **	0.69
Number of obs = 351	Non-selected = 52	Wald chi2(14) = 40.65		Prob > chi2 = 0.0004	

Note: Coef. = estimated coefficient. *, **, ***, the coefficients are significant at the probability levels of 10%, 5%, and 1%, respectively.

## Data Availability

The data presented in this study are available on request from the corresponding author.
